# UNDERSTANDING NEEDS AND CHALLENGES OF HEALTH SELF-MANAGEMENT ACTIVITIES FOR OLDER ADULTS WITH MOBILITY LIMITATIONS

**DOI:** 10.1093/geroni/igz038.1279

**Published:** 2019-11-08

**Authors:** Qiong Nie, Qiong Nie, Wendy Rogers

**Affiliations:** 1 University of Illinois Urbana-Champaign, Illinois, United States; 2 University of Illinois, Champaign, Illinois, United States

## Abstract

According to the U.S. Census, about 30% of older adults live with a mobility impairment, defined as difficulty walking and/or climbing stairs. Older people with mobility limitations are at risk for decreased access to medical services and negative health outcomes. However, few studies address the healthcare needs of these individuals. We used a combination of a population-based data set and an in-depth interview study to understand the challenges these older adults experience in managing medications, accessing health information, going to healthcare provider appointments, and getting help in case of emergency. The quantitative data were from 2,828 participants in the National Health & Aging Trends Study (NHATS). The qualitative data were from 60 participants in the Aging Concerns, Challenges, and Everyday Solution Strategies (ACCESS) interview study. These data provide insights about health self-management challenges for older adults with mobility impairments and guidance for support solutions and interventions.

